# Reducing electric-field-enhancement in metal-dielectric grating by designing grating with asymmetric ridge

**DOI:** 10.1038/s41598-018-22479-3

**Published:** 2018-03-27

**Authors:** Junming Chen, Haopeng Huang, Yibing Zhang, Yonglu Wang, Fanyu Kong, Yanzhi Wang, Yunxia Jin, Peng Chen, Jiao Xu, Jianda Shao

**Affiliations:** 1 Key Laboratory of Materials for High Power Laser, Shanghai Institute of Optics and Fine Mechanics, Chinese Academy of Sciences, Shanghai, 201800 China; 2 University of Chinese Academy of Sciences, Beijing, 100039 China

## Abstract

Diffraction gratings are an essential optical component of high-power, short-pulse lasers. The maximum output of high-power pulsed lasers is always determined by laser resistance of gratings and this resistance is strongly dependent on the local near electric field intensity in the grating structure. We presented a novel method of reducing electric-field-enhancement in metal-dielectric grating by designing asymmetric grating ridge while maintaining high diffraction performance. Compared with the common isosceles trapezoidal grating, the grating with asymmetric ridge got a 0.04% reduction of diffraction efficiency in TE polarization at 1053 nm incident wavelength but a 21.3% reduction of maximal electric-field-enhancement in grating structure. This method can be applied to any surface-relief gratings to reduce the electric-field-enhancement for improving the laser induced damage threshold (LIDT) of grating and supporting the grating-based chirped pulse amplification (CPA) system to develop into higher peak-power levels.

## Introduction

The pulse compression grating (PCG) is one of the most critical components of a high-power grating-based chirped pulse amplification (CPA) laser system^1^, which is an important method for improving the short-pulse-laser output intensity^2^. Metal gratings were firstly and commonly used as PCGs in CPA systems because metals can naturally reflect light with broadband high efficiency^3^, but they were limited to the laser induced damage threshold (LIDT)^2^. Compared with metal gratings, all-dielectric gratings feature negligible losses and much higher LIDTs^2,4,5^ but cannot fulfill the broader bandwidth specifications^6^ and the mechanical stress will increases significantly when the number of layers and size become large^7^. For avoiding these disadvantages mentioned above, mixed metal-dielectric grating with high diffraction efficiency (DE) and broad bandwidth is a viable candidate for the realization of PCG with high LIDT^8^.

**Figure 1:**
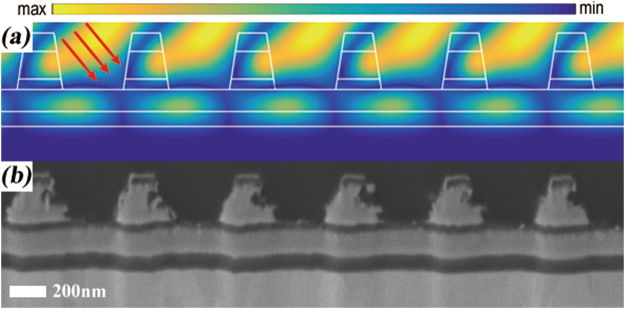
(**a**) Enhancement of the electric field intensity E^2^/E_0_^2^ of the sandwich-ridge grating and (**b**) the cross-sectional view of the damaged grating by scanning electron microscope (SEM). Red arrows represent the incident direction.

A broadband metal-dielectric grating with a sandwich-ridge was designed and fabricated in our previous work^9^ and recently we found that this grating was initially damaged at the grating ridge by femtosecond laser in transverse-electric (TE) polarization. The initial damage was occurred at the location where the enhancement of the electric field intensity (normalized by E^2^/E_0_^2^) is highest in the grating as shown in Fig. 1. This result implied that the femtosecond laser induced damage of the metal-dielectric grating was considered to exhibit a strong dependence on the local near electric field intensity in the grating structure, which was proved by J. Neauport *et al*.^5^. Steve Hocquet *et al*. also reported this phenomenon in multilayer dielectric (MLD) grating and they improved the LIDT from 2.9 J/cm^2^ up to 4.8 J/cm^2^ in normal beam by illuminating the MLD grating from in TE to transverse-magnetic (TM) polarization with a reduction of electric-field- enhancement from 1.38 to 1.16. But they stressed that MLD grating have been designed so far to be used in TE polarization because the DE is much lower in TM polarization^10^. So polarization conversion seems not a practical method and direct reducing electric-field-enhancement in grating is required to improve the LIDT of the grating used with femtosecond laser. Bonod and Neauport reported a drop of the maximum field intensity by increasing the groove width^11^, but Liu *et al*. reported a similar method but decreasing the groove width^12^, so the method of adjusting the groove width seems to be uncommon because it strongly relies on the specific grating itself. In any case, achieving high LIDT while maintaining high diffraction performance is a significant and challenging work for supporting the grating-based CPA system to develop into much higher peak-power levels. In this paper, we present a metal-dielectric grating solution of reducing electric-field-enhancement in the structure and achieving high DE at the same time.

## Results and Discussion

### Impact of the geometrical shape of the grating ridge

**Figure 2:**
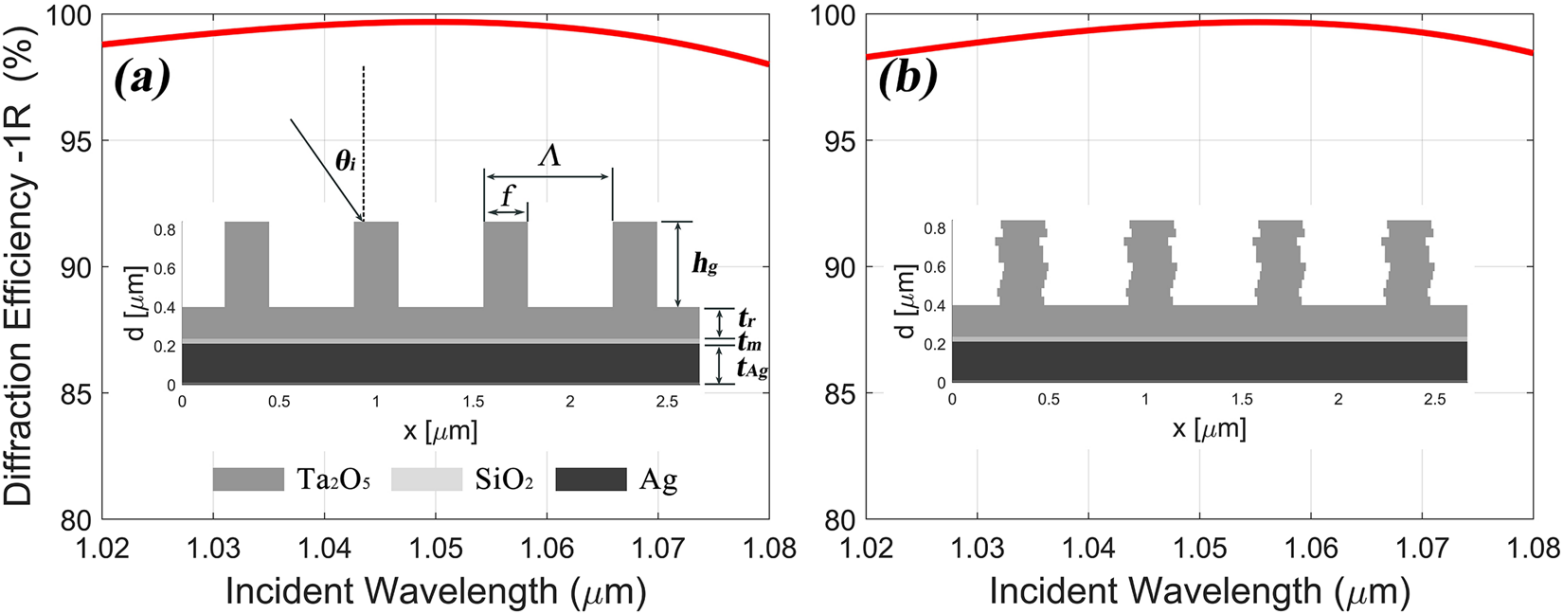
The calculated -1^st^ reflective DE spectra in TE polarization and the schematic structures of the gratings with (**a**) rectangle grating ridge and (**b**) deformed ridge shape by randomly shifting the sliced slabs.

The asymmetric distribution of electric field intensity (see Fig. 1) is resulted from the asymmetric working situation with a non-zero incident angle. So our basic idea is to design specific grating with asymmetric shape to modulate the distribution of electric field intensity in the grating while maintaining high DE. Before doing this, we started from designing a regular grating as an example to justify our method. A common metal-dielectric grating consists of a high reflective metal layer, some phase-matching dielectric layers and the grating ridge^9^. For the sake of simplicity, the grating ridge with single material was set to be rectangle in the beginning. So our grating contains a rectangle grating ridge (height h_g_), a residual layer (thickness t_r_), a matching layer (thickness t_m_), a high reflective Ag layer (thickness t_Ag_) and the substrate. The material of the grating ridge and the residual layer is Ta_2_O_5_ because high-refractive-index material can lower the etching depth and increase the DE and also can be compared to the material in sandwich grating structure mentioned in Fig. 1. The residual layer is added to increase the fabrication tolerance of grating. The matching layer with low-refractive-index material SiO_2_ is set to match the phase for achieving higher DE. We focus on designing the grating with broadband high DE around 1053 nm wavelength. Multi-parameter optimization is carried out in our design because multiple parameters of the grating influence the DE and bandwidth of grating. These parameters included the period of the metal-dielectric grating Λ, the duty cycle f, the height of grating ridge h_g_ and all the thicknesses (t_r_, t_m_ and t_Ag_). The incident angle was fixed at -1^st^ reflective Littrow-mount constraint by $\theta =\arcsin (\lambda /2{\rm{\Lambda }})$ in 1053 nm wavelength for achieving higher efficiency. We designed our grating optimization process and the details of it are described in the methods section. After the optimization process, we got the optimized parameters of the rectangle metal-dielectric grating: Λ = 0.667 μm, f = 0.344, h_g_ = 0.439 μm, t_r_ = 0.163 μm, t_m_ = 0.025 μm and t_Ag_ = 0.2 μm. The calculated -1^st^ reflective DE spectrum in TE polarization and the schematic structure of the grating are shown in Fig. 2(a). This metal-dielectric grating with rectangle ridge came with broadband high efficiency and the DE in 1053 nm central wavelength reached 99.67%. We want to know whether and how the distortion of grating ridge influences the DE of the grating. A basic idea for analyzing the surface-relief grating with arbitrary grating ridge shape is to divide the grating ridge into a large number of thin planar slabs perpendicular to the normal to the boundary^13^. So we sliced the rectangle ridge into 10 thin planar slabs and these slabs were randomly shifted with a slight displacement away from the center. The calculated DE spectrum and the schematic structure of the deformed grating are shown in Fig. 2(b), which presented similar performance with the rectangle grating. This result shows that restricted distortion of grating ridge will not influence much on the DE, so we can design unusual shape of grating based on this result. Compared with the slightly changed DE of grating, the electric-field-enhancement will increase or decrease based on the specific distortion of grating ridge and the direction of distortion plays essential role in the modulation of the electric field intensity in the grating. The details of electric field intensity will be discussed in the next section.

### Design and performance of symmetric and asymmetric gratings

**Figure 3:**
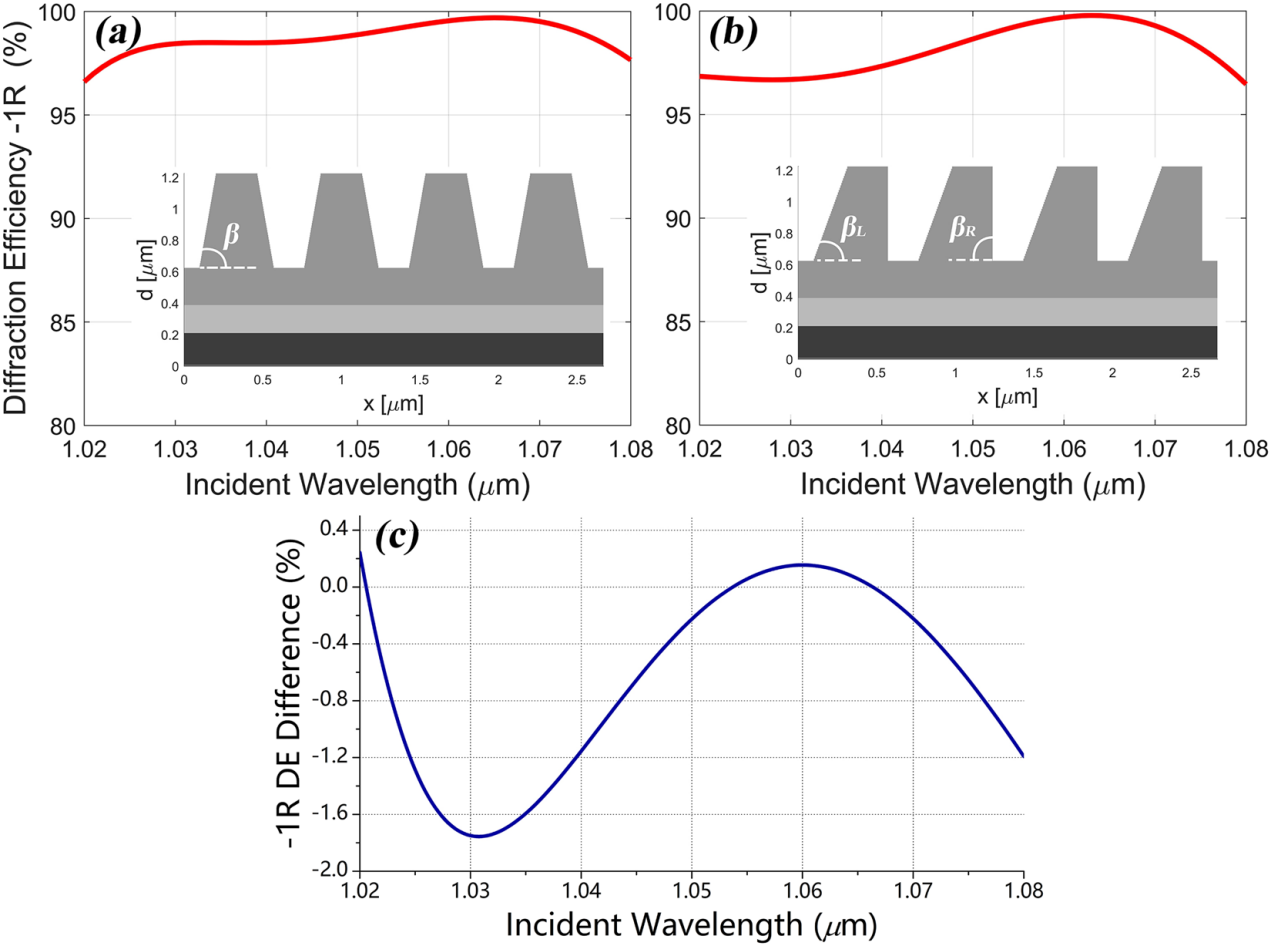
The calculated -1^st^ reflective DE spectra in TE polarization and the schematic structures of the gratings with (**a**) isosceles trapezoidal grating ridge with base angle β = 80° and (**b**) asymmetric trapezoidal grating ridge with two base angles β_L_ = 70° and β_R_ = 90°. (**c**) The calculated -1^st^ reflective DE difference spectrum between the symmetric and asymmetric grating over the considered wavelength range.

Design of isosceles trapezoidal grating ridge with base angle of β rather than the rectangular shape can lower the difficulty of fabrication based on our previous works^9,14,15^. So we re-optimized the metal-dielectric grating into isosceles trapezoid shape to go a step further for describing our designing method. Based on our previous experiments^9,14,15^, the base angle of β in the grating was mostly measured near 80° with a ±10° range, so the isosceles base angle of β was set to 80° for convenience. The period of grating Λ was fixed at 0.667 μm as the same as the rectangle one for keeping consistent working situation. The trapezoidal grating ridge was sliced into 40 slabs to increase the accuracy of simulation. The re-optimized parameters of the isosceles trapezoidal metal-dielectric grating were: f = 0.72, h_g_ = 0.59 μm, t_r_ = 0.236 μm, t_m_ = 0.178 μm and t_Ag_ = 0.2 μm. The calculated DE of the -1^st^ order reflected spectrum in TE polarization and the schematic structure of this grating are shown in Fig. 3(a). The DE at 1053 nm central wavelength reached 99.08% as similar as the rectangle design.

**Figure 4:**
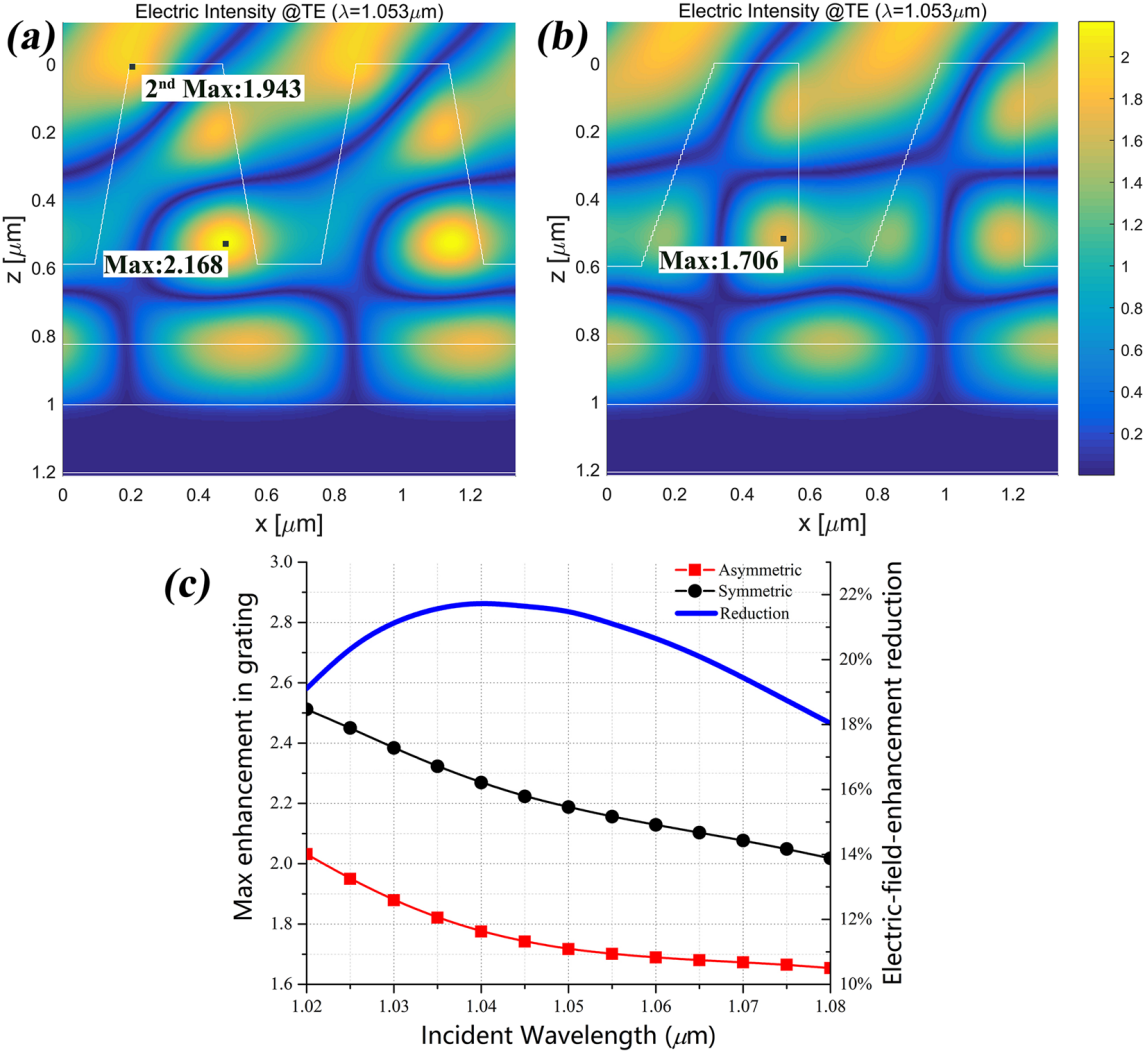
The enhancement of the electric field intensity in TE polarization at 1053 nm in the gratings with (**a**) isosceles trapezoidal grating ridge with a maximum value of 2.168 and (**b**) asymmetric trapezoidal grating ridge with a maximum value of 1.706. (**c**) The calculated maximal enhancement of the electric field in symmetric and asymmetric gratings (left axis) and the electric-field-enhancement reduction spectra (right axis).

We want to design specific asymmetric grating shape to modulate the distribution of electric field intensity of the grating while maintaining high DE. According to the result from Fig. 2, the slabs of the isosceles trapezoidal grating ridge can be slightly shifted a small distance away from center without much reduction in DE. Considering the initial damage has occurred at the grating ridge opposite to the incoming wave (Fig. 1), so we design the grating with non-isosceles trapezoidal ridge and the ridge is slanted opposite to the incoming wave. Two base angles (β_L_ and β_R_) were added to describe our grating shape and they were set to be 70° and 90° respectively when the incident light irradiated from left top. The two angles 70° and 90° are the results from the optimization process, which are separated ±10° from 80° and this equal difference will cause the ridge distortion in a certain direction. Compared with the isosceles trapezoidal grating ridge with 80° base angle, the non-isosceles grating can be considered that the sliced slabs were integrally shifted a small displacement opposite to the incoming wave. The other parameters of the asymmetric grating were optimized with slight changes: f = 0.7, h_g_ = 0.6 μm, t_r_ = 0.23 μm. The calculated -1^st^ reflective DE spectrum in TE polarization and the schematic structure of this asymmetry grating are shown in Fig. 3(b). We found that its DE in 1053 nm central wavelength was 99.04% with a 0.04% reduction compared with the isosceles grating. We have tested the combination of 75° and 85° and the results showed less both DE and electric field reduction. That means larger distortion will result more modulation of the enhancement of the electric field intensity and more reduction of DE at the same time. The combination of 70° and 90° got a balance between the DE and enhancement of the electric field intensity. The maximum and average reduction of DE between the symmetric and asymmetric grating (calculated by ${{\rm{DE}}}_{asym}-{{\rm{DE}}}_{sym}$) over the considered wavelength range are respectively −1.75% and −0.65% as shown in Fig. 3(c). But the distribution of electric field intensity of these two gratings presented much more differences as shown in Fig. 4. Compared with the maximum enhancement (value:2.168) of the electric field intensity in the isosceles trapezoidal grating, that value of the asymmetry grating is 1.706, which means that a 21.3% reduction of electric-field-enhancement in metal-dielectric grating has been achieved. Besides, the obvious enhancement (value:1.943) of electric intensity in the left-top corner of the isosceles trapezoidal grating has been avoided in the asymmetry grating. This reduction not only happened at 1.053 μm but also around the bandwidth with high DE from 1.02 μm to 1.08 μm. As shown in Fig. 4(c), the maximum enhancement of the electric field intensity gradually decreases in both symmetric and asymmetric gratings when the incident wavelength increases, and the reduction exceeds 18% all around the spectrum. According to the study by Steve Hocquet *et al*., they got a 39.58% increasement of LIDT by a 18.96% diminution of electric-field-enhancement^10^, so we have confidence to believe that the LIDT of the metal-dielectric grating can be improved by designing asymmetric grating ridge because of these obvious reductions of electric-field-enhancement in grating.

## Methods

### The optimization process

The optimization is obtained by the genetic algorithm (GA)^16^ to find minimum of merit function and the merit function was taken as a mean-error expression around the considered wavelength range ${\rm{MF}}=\frac{1}{N}{\sum }_{{\lambda }_{i}}^{{\lambda }_{N}}[100 \% -D{E}_{-1R}({\lambda }_{i})+{E}_{Max}({\lambda }_{i})]$, where *N* is the number of wavelength discrete points and ${E}_{Max}$ is the maximum enhancement of the electric field intensity in the grating. Grating parameters will be optimized by this expression to achieve high DE as close as possible to 100% and low the electric field intensity as much as possible. These two goals were considered equally in this merit function. Rigorous coupled-wave analysis^17^ was employed to calculate the DE and the distribution of the enhancement of the electric field intensity. The grating bandwidth can be optimized depended on the wavelength range in the merit function. Using evolutionary algorithms such as GA has been proved to be a straightforward and efficient method to optimized grating based on our previous works^9,15,18^ and GA is also widely used in fiber optics^19^, three-dimensional printings^20^ and other optical components designing^21^.

### Fabrication and damage test

The metal-dielectric grating mentioned in Fig. 1 was fabricated by high power laser coatings, lithography and ion-beam etching technology^9^. In coating process, a chromium film was deposited on the substrate first to enhance the adhesion of metal film and substrate, and all layers were deposited by electron beam evaporation. The coating sample was annealed at 350 °C in air to release internal stress for avoiding delamination in the cleaning process. In lithography process, the grating ridge pattern of photoresist was generated from the interference of two monochromatic collimated beams of ultraviolet (UV) light. In etching process, the grating structures were transferred from the photoresist to the dielectric layers by reaction ion beam etching technology and cleaned by hydrochloric/peroxide mixture combined with mega-sonic cleaning method. Besides, the asymmetric grating ridge can be fabricated by specific slant-etching technology, which is common in slanted grating fabrication based on the studies by Tapani Levola *et al*.^22^ and Kalle Ventola *et al*.^23^. In the damage test procedure, each test site was only exposed to a single shot, and different sites were irradiated with the different laser fluence. The fluence was gradually decreased until the damage didn’t happened. The irradiated sites were preliminarily judged to be damaged or not with the aid of charge-coupled device (CCD) and the details of damaged spots were carried out by SEM.

### Data availability statement

All data generated or analysed during this study are included in this published article.

## Conclusions

In this paper, we presented a novel method of reducing electric-field-enhancement in metal-dielectric grating by designing asymmetric grating ridge. We firstly demonstrated that restricted distortion of grating ridge will not influence much on the DE, which means that grating can be designed with unusual shape while maintaining high DE. Associated with the initial damage at the grating ridge where the enhancement of the electric field intensity is highest in the structure, we modified our optimized isosceles trapezoidal metal-dielectric grating into non-isosceles shape with two base angles for modulating the distribution of electric field intensity in the grating. Compared with the isosceles trapezoidal grating, the grating with asymmetric ridge got a 0.04% reduction of DE in TE polarization at 1053 nm central incident wavelength but a 21.3% reduction of electric-field-enhancement in grating structure. This result indicated that it’s valid to design specific asymmetric grating shape to modulate the distribution of electric field intensity of the grating while maintaining high DE. Asymmetric gratings can be not only used to reduce laser damage but also used for other applications such as near-to-eye display technology and waveguide technology^24–26^. This design method can be applied to any surface-relief gratings to reduce the electric-field-enhancement for improving the LIDT of grating and supporting the grating-based CPA system to develop into much higher power levels.
